# Beef-on-Dairy Crossbreeding: A Multidimensional Analysis and Gaps for Pasture-Based Production Systems

**DOI:** 10.3390/ani16213496

**Published:** 2026-11-05

**Authors:** Héctor A. Uribe, Alejandro Velásquez, Rodrigo Arias

**Affiliations:** 1 Institute of Animal Production, Faculty of Agricultural and Food Sciences, Universidad Austral de Chile, Valdivia 5090000, Chile; hector.uribe@alumnos.uach.cl; 2 Graduate School, Faculty of Agricultural and Food Sciences, Universidad Austral de Chile, Valdivia 5090000, Chile; 3 Research Nucleus in Food Production, Universidad Católica de Temuco, Manuel Montt 56, Temuco 4780000, Chile; avelasquez@uct.cl; 4 Department of Agricultural Sciences and Aquaculture, Faculty of Natural Resources, Universidad Católica de Temuco, Temuco 4780000, Chile; 5 Volcanic Soils Center, Universidad Austral de Chile, Valdivia 5090000, Chile

**Keywords:** beef × dairy, calving ease, carcass merit, feed efficiency, genomic evaluation, pasture-based systems

## Abstract

Beef-on-dairy (BoD) crossbreeding, defined as the planned use of beef sires on dairy dams, has expanded as a strategy to increase the value of dairy-origin calves while maintaining herd replacement requirements. This review critically synthesizes research published since 2016 across six interconnected dimensions: genetics and genomics, management and productive efficiency, carcass performance and meat quality, farm economics, environmental sustainability, and animal welfare. The available evidence indicates that BoD can increase calf market value and improve selected growth and carcass traits, although the magnitude of these effects varies with sire genetic merit, dairy breed, reproductive performance, calf management, and finishing conditions. Genetic selection involves balancing calving ease and other functional traits against postnatal growth and carcass merit. Economic outcomes depend not only on calf prices but also on semen costs, reproductive efficiency, replacement requirements, and rearing and finishing expenses; therefore, improvements in individual production traits do not necessarily translate into greater whole-farm profitability. Environmental performance is similarly context-dependent and requires explicit consideration of system boundaries, functional units, emissions intensity, and potential soil carbon sequestration. Animal welfare must also be evaluated throughout the production chain rather than inferred from productive performance alone. A major limitation of the available evidence is its concentration in intensive and semi-intensive production systems, which restricts its transferability to permanent-pasture environments. Further research should prioritize longitudinal, farm-level evaluations from birth to slaughter under defined pasture-based conditions, integrating genetic and management decisions with productive, economic, environmental, and welfare outcomes.

## Simple Summary

Dairy farmers increasingly use beef bulls on dairy cows to produce calves for beef production while using dairy or sexed semen to obtain replacement heifers. This practice, known as beef-on-dairy (BoD) crossbreeding, can increase the value of calves born in dairy herds. This review examines scientific studies published since 2016 to evaluate BoD across genetics, animal management, growth, carcass and meat quality, farm economics, environmental sustainability, and animal welfare. The evidence shows that beef-sired calves can achieve higher market values and improvements in selected carcass traits, although the magnitude of these benefits varies with sire genetics, reproductive performance, calf management, and finishing conditions. Greater calf value or heavier carcasses do not necessarily result in higher farm profits, because semen, rearing, finishing, and replacement costs must also be considered. Environmental outcomes likewise depend on how production systems are managed and assessed, while animal welfare requires attention throughout the production chain. Most available evidence comes from intensive or semi-intensive systems, making it difficult to determine whether similar outcomes can be expected under permanent-pasture conditions. Further research should follow animals from birth to slaughter in pasture-based systems and evaluate productive, economic, environmental, and welfare outcomes together.

## 1. Introduction

Beef-on-dairy (BoD) crossbreeding, the planned mating of beef sires to dairy dams to produce calves for beef rather than for herd replacement, allows dairy producers to add value to non-replacement calves while maintaining replacement heifer supply. Its economic value, however, is not determined by calf sale price alone: calving performance, calf survival, postnatal growth, carcass merit, and rearing and finishing costs all contribute. Variation among beef sire breeds can be substantial. In Irish national records, Berry et al. [1] reported a €125.4 difference in mean calf sale price between the highest- and lowest-valued beef breeds, and mean steer carcass weight ranged from 327.1 kg for Angus-sired to 363.2 kg for Belgian Blue-sired progeny. Neither figure establishes a net economic advantage, which depends on the balance among revenue, costs, animal losses, and the performance of the whole system.

The outcomes of BoD systems reflect the interaction of sire genetic merit, dam characteristics, and management rather than sire breed alone [2,3,4]. Sire selection entails a trade-off between reproductive traits—birth weight, gestation length (GL), and calving ease (CE)—and postnatal growth and carcass merit [2,5,6,7]. Breed averages also conceal variation among individual bulls, and rearing, nutrition, grazing, and finishing practices determine how much genetic potential reaches the saleable animal [3,8]. BoD effects are therefore conditional on the genotype-by-environment combination in which they are expressed, which is why single-trait or breed-level rankings are weak guides to whole-system performance.

Evidence on reproduction, genetic evaluation, growth, and carcass merit has accumulated rapidly, but it does not amount to an integrated assessment. Studies differ in population, production environment, management, trait definitions, and analytical method, which limits direct comparison. Recent work has addressed the genetic evaluation of crossbreds and genomic variation in economically relevant traits [9,10], yet neither breeding values nor animal-level performance measures establish the commercial value of a strategy. An improvement in a single performance trait may fail to raise overall value when it carries higher costs, greater losses, or welfare costs, so quantitative evidence from different dimensions needs to be read together, with explicit attention to the uncertainty of the underlying studies.

Transferability is a second constraint. Feed availability, diet composition, grazing management, and finishing strategy alter growth trajectories, slaughter age and weight, carcass composition, and cost structure, and in temperate grassland systems the cost of feed production and the contribution of grazed pasture are central to the economics of dairy calf-to-beef production [11,12]. Nutritional and finishing decisions further modify the expression of productive potential [3]. For pasture-based dairy regions such as southern Chile, where permanent pasture underpins livestock production, the relevance of evidence generated in confinement systems is a question to be tested rather than assumed.

This review synthesizes research published since 2016 across six dimensions: genetics and genomics; management and productive efficiency; carcass merit and beef quality; economics; environmental sustainability; and animal welfare (Figure 1). Emphasis is placed on quantitative findings—sample sizes and effect magnitudes for reproductive, growth, carcass, and economic outcomes—together with the populations, production systems, and methodological limits behind each result. Three questions organize the analysis. First, which BoD effects are sufficiently consistent across studies to be treated as established? Second, which depend on sire, dam, management, or market conditions and therefore cannot be generalized? Third, which findings can be transferred to temperate permanent-pasture systems, and where is locally relevant evidence missing? Distinguishing established findings from context-dependent ones and from open questions is the basis on which this review derives implications for research and for the design of BoD breeding and management strategies.

## 2. Genetics and Genomics of the Beef × Dairy Cross

### 2.1. Heterosis and Breed Complementarity

Two related but distinct mechanisms underlie BoD crossbreeding. Heterosis is the deviation of crossbred performance from the mean of the parental populations and reflects non-additive genetic effects; breed complementarity is the combination of favorable additive attributes of different breeds to meet a defined production objective [2,13]. In BoD systems the aim is to retain the reproductive and maternal function of the dairy dam while improving growth and carcass traits in the terminal progeny. Both mechanisms contribute to traits such as birth weight, preweaning growth, and weaning weight, but their relative magnitude varies with trait and breed combination [14]. Maternal heterosis, the effect of the crossbred dam on her offspring, is a separate component; Knapp et al. [15] estimated maternal heterosis of 1.0% for average daily gain (ADG) from birth to weaning and 1.1% for 205-day weaning weight, although those estimates came largely from conventional beef crosses, including a small group of beef × Brown Swiss calves, and do not transfer directly to contemporary BoD populations.

In practice, breed complementarity frames the choice of beef sire around the intended balance between calving performance, growth, and carcass value. Breed averages, however, do not represent the merit of individual bulls, and differences among sire-breed groups also reflect within-breed selection, dam characteristics, and management. Neither heterosis nor complementarity therefore establishes a universally superior beef breed: their value depends on the breeding objective, the merit of individual sires, the dairy dam population, and the production environment. Section 2.3 examines the resulting trade-off between calving performance and carcass value.

### 2.2. Genomic Tools for Optimizing Beef-on-Dairy Systems

Genomic evaluations are built in purebred reference populations, whereas the breeding objective in BoD is crossbred progeny performance. Differences in linkage disequilibrium, allele frequency, and the expression of genetic effects reduce the transferability of predictions from purebred candidates to crossbred animals. The relative advantage of purebred and crossbred reference populations depends on the genetic correlation between purebred and crossbred performance, the relatedness between reference animals and selection candidates, and reference population size [16,17].

Direct evidence from BoD populations is limited but informative. Mugambe et al. [9] analyzed 4420 BoD calves from 224 farms in Schleswig-Holstein (2017–2023), sired by Angus, Limousin, Wagyu, or White Belgian Blue bulls out of Holstein dams. A bivariate model raised prediction accuracy for CE to 0.22 from 0.13 in the univariate model, an absolute gain of 0.09, whereas accuracy for GL (0.32 vs. 0.31) and birth weight (0.32 vs. 0.30) was essentially unchanged. The gain in CE is consistent with information shared through genetic correlations, but the accuracy estimates alone do not show how much independent predictive value the additional trait contributed, and they do not establish that a crossbred reference population outperforms a purebred one.

Heritability estimates from BoD populations indicate the scope for genetic change. Ahmed et al. [10] reported heritabilities of 0.29 (±0.03) for birth weight, 0.36 (±0.04) for calving difficulty, and 0.09 (±0.03) for GL in Angus- and Belgian Blue-sired calves out of Holstein dams, and genome-wide analyses that included dominance effects identified an additional region associated with birth weight. Genome scans have also begun to describe the architecture of crossbred performance: Lindtke et al. [18] associated variants in the FAM184B–NCAPG–DCAF16–LCORL region with carcass weight and protein mass in Brown Swiss crosses, and reported lower genomic inbreeding in crossbred than in purebred cows (0.381–0.392 vs. 0.461). Such associations require validation in independent populations before they can support selection decisions.

The practical value of genomic tools in BoD therefore rests on the size and representativeness of reference populations, the accuracy achieved in the target crossbred population, and validation across dairy breeds and environments. For South American pasture-based systems, the transferability of evaluations developed in European populations remains an open question, and the contribution of genomic selection to commercial performance has yet to be demonstrated under those conditions.

### 2.3. Calving Ease Versus Carcass Merit: The Central Genetic Trade-Off

The central genetic trade-off in BoD is between the carcass value of terminal progeny and the calving risk borne by the dairy dam. Beef breeds differ in growth potential, GL, and calving performance, but breed rankings conceal substantial variation among individual sires. Berry et al. [1], using up to 977,037 calving records and 103,175 carcass records, reported mean GL for male calves of 282.3 days for Angus, 287.4 days for Limousin, and 280.9 days for Holstein-Friesian sires—a 5.1-day range with direct consequences for calving management. The same records showed odds ratios for dystocia, relative to Holstein-Friesian sires, of 1.43 for Angus and 4.77 for Belgian Blue before adjustment for maternal and direct genetic merit for calving difficulty. Adjustment attenuated the breed differences, which is the clearest demonstration that breed averages and individual genetic merit are not interchangeable.

Carcass outcomes in the same records show why carcass weight and dressing percentage (DP) must be distinguished. Mean carcass weight was 327.1 kg for Angus-sired, 363.2 kg for Belgian Blue-sired, and 322.4 kg for Holstein-Friesian-sired steers, a 40.8 kg advantage for Belgian Blue over Holstein-Friesian. These differences cannot be attributed to DP without corresponding slaughter weights. Finishing system also matters: Maciel et al. [19] reported DP of 57.6% in grazing-finished and 61.9% in grain-finished steers, a difference of 4.3 percentage points, although these values cannot be extrapolated directly to BoD populations.

Such descriptive differences reflect the production and slaughter conditions represented in the records as much as breed genetics, and they show why carcass weight cannot serve as the sole selection criterion: greater carcass output may be obtained at the cost of higher calving risk, depending on sire merit and on the females bred.

The German dataset of Ahmed et al. [6] (3966 calves, 122 farms) shows the same pattern from the calf side: Wagyu-sired calves had the lowest and Limousin-sired calves the highest observed calving difficulty, male calves had 1.94 times the odds of difficult calving compared with female calves, GL averaged 280.2 days for Angus- and 287.6 days for Limousin-sired calves, and heifers calved with more difficulty than multiparous cows. Unequal and small breed-group sizes, particularly for Limousin (*n* = 169) and Wagyu (*n* = 145), limit breed comparisons. The practical response to this trade-off has been to rank bulls on a multi-trait index rather than on a single trait. Berry et al. [5] developed a dairy-beef index combining calving traits and beef performance in one economic objective, and Berry and Ring [20], using 123,785 calving records and carcass data from 48,875 animals, showed that progeny of higher-index bulls had heavier and better-conformed carcasses than progeny of bulls selected mainly for calving performance, with an estimated 3–5% increase in dairy-herd profit under the assumptions of that study. That figure is population- and assumption-dependent and is not a guaranteed return elsewhere, but it shows that calving and carcass traits can be balanced within a single selection objective.

For BoD mating decisions, the implication is that individual-bull evaluations for CE and GL, where available, carry more information than breed averages, and that the acceptable balance between calving risk and carcass merit depends on dam parity and size, sire merit, market endpoint, and the relative costs of dystocia and of carcass gain.

## 3. Management and Productive Efficiency

### 3.1. Production Systems and Management Strategies

Management determines how far the genetic potential of BoD cattle is expressed in growth, carcass characteristics, and net return, and the decisions taken before weaning, after weaning, and during finishing are interdependent rather than separate interventions. Evidence for individual practices also differs in strength, and short-term responses cannot be read as persistent effects on carcass value. During the preweaning period, milk allowance and nutrient density modify early growth and muscle development: Carter et al. [21] fed 42 SimAngus × Holstein calves milk replacers containing 30% crude protein and 32% fat or 22% protein and 20% fat to 8 weeks of age, and the higher-nutrient treatment increased body-weight gain and muscle-fiber development. The study was small and ended in early life, so it does not show that these responses persist to slaughter.

Stypinski et al. [22] linked early nutrition more directly to finishing performance in 40 Angus × Holstein calves given milk replacers with 20% or 24% crude protein and fat; over 56 days the higher-nutrient treatment increased ADG by 23.6% and gain-to-feed ratio (G) by 9.8%. Neither study followed animals through finishing, and both samples were small, so the persistence of the advantage, and its interaction with feed cost, calf health, and the weaning transition, remain untested.

The longest follow-up available indicates effects that persist but are not uniform across traits. Grote et al. [23] assigned 109 beef × dairy steers to higher or moderate nutrient programs to 92 days of age and finished them for 308 days. Steers on the higher-nutrient program remained heavier through day 168 of finishing and produced a greater proportion of United States Department of Agriculture (USDA) Choice carcasses, whereas the moderate-nutrient group had a greater G during part of finishing and overall. No other carcass trait differed, and the moderate group showed no compensatory growth sufficient to erase the early weight disadvantage. Early nutrition therefore influences later performance, but heavier body weight does not imply better feed efficiency across the cycle.

After weaning, the choice between a pasture-based growth phase and direct feedlot entry sets the balance among forage use, growth rate, finishing duration, and carcass development. A pasture phase can lower the cost of gain where forage is available, but its outcome depends on forage quality, seasonality, stocking rate, supplementation, and the finishing strategy that follows; feedlot entry offers a more controlled nutritional environment at the cost of purchased feed and closer management. Neither route is superior in general, and comparisons require common endpoints and total system costs.

Commercial-scale evidence shows how much this variability matters. Grote et al. [24] compared 184 beef × dairy with 160 native beef steers under calf-fed and yearling-fed systems, the latter grazing mixed-grass prairie for 144 days before feedlot entry. Beef × dairy steers in the yearling-fed system had the greatest overall ADG, consistent with compensatory growth, and total returns from beef × dairy systems were intermediate between the two native-beef systems. Interpretation is limited because each breed-type × finishing-system combination occupied a single commercial pen, so pen effects cannot be separated from treatment effects. Liver abscesses were also far more frequent in beef × dairy (82.5%) than in native steers (37.3%), and more frequent in yearling-fed (70.3%) than in calf-fed animals (54.2%)—a health-management signal that cannot be attributed to grazing itself, since breed type, finishing management, and pen conditions may all contribute.

Breeding decisions are therefore inseparable from rearing and finishing management. Calving ease and calf survival determine how many animals reach the beef chain, while growth rate, feed efficiency, days to slaughter, and carcass composition determine finishing cost and saleable output. These outcomes have to be assessed jointly: extending finishing may add carcass weight or quality, but the additional feed, labor, and health costs can outweigh the gain, and a faster growth rate does not imply greater profitability if it brings higher feed or health expenditure, or carcasses outside the payment grid.

### 3.2. Optimizing Days on Feed

Days on feed (DOF) influences final body weight, carcass weight and quality, feed efficiency, and the cost of maintaining animals until slaughter. Extending finishing can raise carcass value, but the gain has to be set against additional feed, housing, health, and capital costs. The relevant objective is therefore the slaughter endpoint that maximizes net return under a given production and market context, not maximum weight or maximum quality in isolation.

Husz et al. [25] assigned 3765 beef × dairy heifers to finishing endpoints of 323, 344, 362, or 385 DOF. Final body weight increased from 593 to 655 kg, and hot carcass weight (HCW), longissimus muscle area, calculated yield grade, and marbling score all increased with longer finishing, whereas dry matter intake (DMI) was unaffected. In contrast, ADG and G declined quadratically, and DP and backfat thickness followed quadratic responses. Across the 62-day difference between the shortest and longest endpoints, HCW increased by approximately 0.67 kg per additional day. Extending finishing therefore adds weight and improves selected carcass attributes, but growth and feed conversion do not remain constant.

Schmaltz et al. [26] translated the same kind of data into net returns and market risk under alternative price and cost scenarios. Under baseline assumptions the shortest finishing treatment gave the highest estimated net return (US$99.84 per head) and the longest the lowest (US$57.13 per head), and the ranking of endpoints changed when feed costs or carcass prices shifted, with carcass price the main source of variation. These values are outputs of a specific economic model rather than profitability predictions, but they show that the endpoint maximizing carcass weight or quality is not necessarily the one maximizing net return.

Crawford et al. [27] evaluated 707 beef × dairy feedlot steers harvested at 326, 347, or 368 DOF. Final body weights were 696, 730, and 744 kg, and HCW rose from 438 to 459 and 472 kg, but ADG from allocation to slaughter fell from 1.82 to 1.64 kg/d and feed efficiency from 0.162 to 0.143, with DMI similar among treatments (approximately 11.4 kg/d). Marbling scores increased with longer finishing, whereas the distribution of USDA quality grades did not differ. As this work was published as a conference abstract, it is complementary evidence rather than a substitute for a full peer-reviewed report.

Taken together, these studies show that DOF involves trade-offs among carcass output, composition, feed efficiency, eating quality, and net return, and that the preferred endpoint depends on sex, initial weight, genetic background, feed costs, payment grid, and market specification. Longer finishing is justified where additional carcass weight or quality premiums exceed the marginal cost, and shorter finishing where marginal gain is limited, feed is expensive, or carcasses risk exceeding target specifications. Tenderness may also respond to finishing duration, although the response depends on genotype, growth rate, and production system [28,29]; in feedlot-finished British and Continental crossbred steers, Van Koevering et al. [29] found that tenderness tended to improve with time on feed while performance and carcass results supported a finishing period of 119–133 days under the conditions evaluated, an interval that is not a universal minimum, least of all under grazing. The relevant comparison is marginal rather than total: whether the additional carcass value and any gain in eating quality repay the cost of the extra days.

For pasture-based BoD systems the slaughter endpoint remains an open question. It may depend on seasonal forage availability, pasture quality, growth achieved at grazing, and whether animals subsequently enter a feedlot; a longer DOF allows greater use of pasture where supply and quality are adequate, but cannot be assumed to improve profitability or environmental performance without direct comparison. The studies above describe intensive finishing and do not establish an optimum for permanent pasture, so pasture-based and mixed pasture–feedlot strategies have to be compared on common measurements of growth, intake, carcass value, total cost, and net return.

### 3.3. Feed Efficiency: Current Evidence and Knowledge Gaps

Feed efficiency is a major determinant of biological and economic performance, but comparisons among studies depend on the metric used: DMI, feed conversion ratio (FCR), gain-to-feed ratio (G), and residual feed intake (RFI) describe different aspects of feed utilization. DMI measures intake; FCR expresses feed required per unit of gain and G its inverse, so that higher G and lower FCR indicate better efficiency; RFI is the difference between observed intake and intake expected from production and maintenance requirements, with lower values indicating greater efficiency. These distinctions matter in BoD systems, where genotypes differ in mature size, growth trajectory, and maintenance requirement, and where the metric and estimation method can rank animals differently [30].

Evidence from intensive finishing suggests that beef × dairy crossbreeding can improve feed efficiency relative to straightbred dairy cattle, although the magnitude varies with study and sire breed. Pimentel-Concepción et al. [31] compared beef × Holstein and straightbred Holstein steers (n = 60 each): beef × Holstein steers had 5% higher numerical ADG (1.75 vs. 1.70 kg/d) with similar DMI (10.40 vs. 10.35 kg/d), 4% higher G (0.172 vs. 0.165), and a lower cost of gain (US$2.68 vs. US$2.83/kg), and reached similar carcass weight despite lower final live weight (365 vs. 367 kg). Under those conditions the crossbred steers converted feed into gain more efficiently without eating more, although the comparison concerns one group of beef-sired Holstein steers in a feedlot.

Rezagholivand et al. [32] evaluated 120 calves from four Holstein-beef crosses (Angus × Holstein, Charolais × Holstein, Limousin × Holstein, and INRA 95 × Holstein) and straightbred Holsteins over an 11-month intensive period. ADG was higher in crossbreds than in straightbred Holsteins (1.35 vs. 1.25 kg/d), but efficiency differed among crossbred groups: Charolais × Holstein calves combined relatively low DMI with an FCR of 6.37, against 8.02 in straightbred Holsteins, and DP and estimated net returns also differed among genetic groups. The response to crossbreeding is therefore not uniform, and sire breed may influence both feed utilization and economic performance, although intensive conditions preclude inference about permanent pasture.

Differences in efficiency cannot be attributed to breed identity alone. Feed utilization reflects digestion, metabolic activity, body composition, physical activity, and thermoregulation [30], so growth rate and intake have to be read alongside body weight, maturity, carcass composition, and production environment. Comparisons of animals slaughtered at different ages or endpoints confound genetic differences with differences in physiological maturity, and individual sire effects contribute variation within the broad BoD category.

Measurement is a further constraint. Feed efficiency is normally recorded under controlled or intensive conditions, where individual or pen intake can be measured; under grazing, individual DMI is difficult to obtain because forage intake varies with pasture availability, herbage quality, grazing behavior, and season. An animal that is efficient in a feedlot does not necessarily retain that advantage at pasture, where locomotion, forage selection, and the energy cost of grazing add to requirements, so feedlot estimates cannot be extrapolated to permanent pasture without validation.

The evidence available supports a narrower conclusion than is sometimes drawn: selected beef × dairy crosses can improve feed efficiency relative to straightbred Holsteins under intensive finishing, but the magnitude and consistency of the advantage depend on genetic background, management, and the metric used. Nothing in the current literature establishes that BoD cattle are intrinsically more efficient across production systems, and least of all under permanent grazing.

## 4. Carcass and Beef Quality

### 4.1. Quantitative Carcass Traits

Carcass weight, dressing percentage, muscularity, fat deposition, and marbling are distinct traits and cannot be treated as interchangeable indicators of carcass merit: longissimus muscle area and subcutaneous fat depth describe muscularity and fatness, whereas marbling describes intramuscular fat and drives quality grading. The value of a sire therefore depends on the combination of traits the target market pays for. Basiel et al. [7] found differences among sire-breed groups in ADG, marbling score, and Warner–Bratzler shear force (WBSF) but not in hot carcass weight (HCW) or DP, whereas Berry et al. [1] documented differences in mean carcass weight in Irish national records (Table 1).

Berry et al. [1] analyzed 103,175 carcass records and reported mean carcass weights of 327.1 kg for Angus-sired, 363.2 kg for Belgian Blue-sired, and 322.4 kg for Holstein-Friesian-sired progeny. These differences suggest that sire selection can influence carcass output, but they have to be read within the population, production system, and statistical model of the original study: breed means do not represent every sire within a breed, and a difference in carcass weight does not imply an equivalent difference in DP, saleable yield, or profit.

Controlled comparisons show that sire-breed effects are more apparent in some carcass attributes than in others. In the 262 beef × Holstein steers evaluated by Basiel et al. [7], sire breed did not significantly affect HCW, DP, backfat thickness, longissimus muscle area, or calculated USDA yield grade: mean HCW ranged from 355 kg in Wagyu-sired to 392 kg in Angus- and Simmental-sired steers, and DP from 60.2% to 61.9%. Marbling, however, differed (*p* < 0.05), with Red Angus highest (5.03), followed by Angus (4.82) and Charolais (4.71), and Limousin lowest (4.14). Composition and quality traits therefore respond differently to sire selection, and the absence of differences in HCW does not imply equivalent performance across economically relevant traits.

Individual sire variation is especially visible under grazing. In a New Zealand progeny test, Martín et al. [33] evaluated carcass records from approximately 1000 beef-cross-dairy animals by 73 Angus and Hereford sires on hill-country pasture: mean progeny-group values were 567 kg slaughter live weight, 277 kg carcass weight, 48.9% DP, and 73.6 cm^2^ longissimus muscle area. Sire effects were significant for carcass size and fat traits but not for fat color, meat color, or ultimate pH, and the difference in mean carcass weight between the best and worst sires was 46 kg—direct evidence that within-breed variation can be substantial. Cross-study comparison nonetheless requires care, because carcass weight depends on slaughter weight, age, sex, finishing duration, and management, and DP depends on the definition of preslaughter weight and on which components are included. Basiel et al. [7], for instance, added an estimated 2.5% for kidney, pelvic, and heart fat because these had been removed before weighing.

A multi-trait selection strategy follows from this evidence rather than selection on breed identity. Expected progeny performance for HCW, muscularity, fat deposition, and marbling has to be considered together with calving traits and market requirements, and within-breed estimated breeding values and terminal indices are a more targeted basis than unadjusted breed averages. The economic weight assigned to each trait will still depend on the payment grid, the market specification, and the cost of achieving the desired phenotype.

For pasture-based systems the open question is whether genetic rankings for carcass traits hold across grazing and intensive finishing, since direct comparisons remain limited by differences in genetics, slaughter endpoint, carcass definition, and management. Standardized carcass measurement and sire-level evaluation across pasture-based, mixed pasture–feedlot, and confined systems would be needed to answer it, and for temperate South American production the answer has to account for pasture use, seasonal growth, and carcass-market requirements together.

### 4.2. Beef Quality and Consumer Value

Quality attributes—intramuscular fat (IMF), tenderness, color, ultimate pH, water-holding capacity, cooking loss, and fatty acid composition—determine consumer acceptance and product differentiation rather than processing yield, and they have to be evaluated separately from carcass weight and muscularity, because improvement in one trait does not imply improvement in the others.

Bittante et al. [34] compared meat quality in 231 calves from different genetic groups and production pathways, including beef × dairy crosses destined for veal or for conventional beef. Quality differed between veal and beef groups, and beef produced on the dairy farm of origin had lower cooking losses and WBSF than beef from specialized fattening units, despite older and lighter animals at slaughter. Management may therefore contribute to quality variation, but differences in animal characteristics, slaughter conditions, and management prevent isolating its independent effect.

Sex effects within a crossbred population were reported by Drachmann et al. [35] in 412 Danish Blue × Holstein rosé veal calves and 183 culled Holstein cows: among crossbred calves, bulls had higher WBSF (51.9 vs. 45.8 N) and cooking losses (11.1% vs. 9.95%) but lower IMF (1.61% vs. 2.92%) than heifers (*p* < 0.001). Tenderness, cooking loss, and fat deposition can therefore differ between sexes within the same cross, although rosé veal production does not represent cattle finished to conventional beef weights.

Sire breed can also affect fatty acid composition. Ivankovic et al. [36] examined 65 beef × Holstein-Friesian bulls sired by Belgian Blue, Piedmontese, Limousin, or Simmental bulls and found a higher proportion of polyunsaturated fatty acids (PUFA) in Piedmontese crosses (20.95% of total fatty acids) than in the other groups (12.88–16.93%; *p* < 0.05). The sample was small and drawn from a single production context, and fatty acid profiles, although used to characterize nutritional composition, do not by themselves demonstrate health benefits in consumers.

The biological basis of these traits differs. Tenderness depends on muscle structure, connective tissue, post-mortem proteolysis, and aging; IMF and fatty acid composition on genetics, diet, and finishing; color on pigment concentration, oxidative status, ultimate pH, and storage. Comparisons therefore require consistent definitions, sampling sites, aging protocols, and analytical methods, and WBSF values are meaningful only in relation to the cooking and sample-preparation protocol used.

The available evidence does not establish that BoD cattle produce consistently superior meat quality to straightbred dairy cattle or conventional beef breeds. Outcomes vary with the trait, genetic background, sex, production conditions, slaughter endpoint, and post-mortem handling, and differences in populations and protocols limit both direct comparison and any universal sire ranking. Much of the evidence also comes from intensive or semi-intensive systems, including rosé veal production.

### 4.3. From Trait Merit to Selection Objectives

Transfer to pasture-based systems requires caution. Temperate humid grazing systems differ in forage quality, seasonal feed availability, growth trajectory, finishing duration, and the relative cost of feed and labor, and these differences can alter both the ranking of genotypes and the economic value of specific carcass traits. The value of additional growth potential, for example, depends on whether cattle can express it from grazed forage or require substantial supplementation, and selection for carcass weight and conformation has to be weighed against calving performance, survival, and the ability to reach specification within the grazing season.

Selection indexes make those weights explicit, and their construction is system-specific. Meier et al. [37] developed economic indexes for beef-on-dairy (B × D) and beef-on-beef (B × B) systems and evaluated 27,123 beef bulls. The average Spearman rank correlation between the two rankings was 0.931, yet only 70.7% of the top 100 sires overlapped, and applying B × B indexes in B × D systems carried an estimated opportunity cost of US$3.24 per mating exposure, with a maximum of US$8.26. Broadly similar rankings can therefore produce economically relevant differences in mating decisions, although these estimates rest on US production assumptions, carcass targets, feeding strategies, and economic weights.

## 5. Economics of Beef-on-Dairy Crossbreeding

### 5.1. Economic Drivers of Beef-on-Dairy Adoption

The economic case for BoD crossbreeding rests on the higher market value of beef-sired dairy calves and on their greater value at slaughter, and it cannot be assessed from carcass or meat-quality traits alone: sire selection, calf rearing, finishing strategy, market specification, and production costs interact across the cycle, and a sire that produces heavier or better-conformed carcasses does not maximize farm profit under every payment scheme. The size of the calf premium also varies. Using records for 79,903 calves in the Irish National Cattle Database, Berry et al. [1] reported a €125.4 difference between the highest- and lowest-valued beef sire breeds, with Charolais-sired calves the most valuable and Angus-sired the least, alongside mean steer carcass weights from 327.1 kg for Angus to 363.2 kg for Belgian Blue, against 322.4 kg for Holstein-Friesian. The price differential cannot be attributed to carcass merit alone, since market conditions and other calf characteristics also influence value.

The incentive to produce higher-value calves has driven the adoption of beef semen: dairy genetics supply the replacement generation while matings from cows not needed for renewal are directed to beef production. The value of the strategy depends on the calf price received and on the costs of semen, reproductive management, and replacement supply, so a higher calf price alone does not make a breeding program more profitable at herd level.

Industry-level change illustrates the scale of the shift and its lag. In the United States, greater use of beef semen alongside sex-sorted dairy semen has altered the balance between beef-crossbred calf production and heifer supply: CoBank [38] projected a combined reduction of approximately 796,000 dairy replacements entering the milking herd during 2025–2026, followed by a recovery of approximately 360,200 head in 2027–2028. These are industry projections based on semen-sales trends and reproductive assumptions rather than observed outcomes, but they show the biological delay between mating decisions and the availability of replacement animals.

### 5.2. Semen Allocation and Selection Indices

The economic value of BoD depends on how conventional dairy, sex-sorted dairy, and beef semen are allocated across heifers and cows, a decision that trades replacement supply against the production of higher-value calves. The optimum depends on herd fertility, replacement requirement, semen prices, calf values, and the reproductive category of each animal rather than on a fixed proportion of beef matings.

Cabrera [39] formalized this trade-off in an income over calf and semen costs (ICOSC) model applied to a simulated 1000-cow Holstein herd, combining five levels of beef-semen use in adult cows with six strategies for sex-sorted semen. The optimal strategy changed with reproductive performance, expressed as the 21-day pregnancy rate—the proportion of eligible cows that conceive within each 21-day interval of the breeding period. At approximately 20%, the highest ICOSC consistent with replacement balance was US$2001 per month, obtained by using sex-sorted semen for the first two services in heifers and the first service in first- and second-lactation cows, with beef semen for the remaining eligible matings. At approximately 30% the maximum rose to US$6215 per month. At approximately 15%, no strategy achieved positive ICOSC and a sufficient replacement supply simultaneously. ICOSC is income over calf and semen costs, not total farm profit, and the estimates depend on the model’s calf prices, semen costs, herd structure, and reproductive parameters.

Clasen et al. [40] combined sex-sorted semen, beef semen, genomic testing, and terminal crossbreeding in simulated Swedish herds and found the largest gains when the tools were used together, with the highest modeled return rising by €58 for Swedish Holstein and €94 for Swedish Red relative to pure breeding without sex-sorted semen or genomic testing. The result matters because strategies that raise short-term calf value can also change the rate of genetic gain in the dairy herd. Farm evidence points the same way: in a California survey of 141 dairy operations, 81% used beef semen and 78% named additional profit as a principal advantage, although the 13.9% response rate means the findings describe the participating farms rather than national adoption [41]. Ferrari et al. [42] reached a similar conclusion in a 350-cow Italian herd, where combining sex-sorted and beef semen reduced replacement costs while maintaining heifer supply, with the optimum again depending on herd fertility and replacement needs.

Selection indices translate these trade-offs into a ranking of bulls. In Ireland the Dairy Beef Index (DBI) combines calving performance, calf mortality, feed intake, carcass weight, conformation and fatness, carcass specification, docility, and polledness in a single objective, so that sires are identified by the beef value of their calves and by the consequences of the mating for the dairy herd. The economic weights embedded in a national index reflect its own production conditions, breeding objectives, and market structure, and have to be reassessed before use elsewhere.

The value of such an index has been quantified with Irish data. Berry and Ring [20] used 123,785 calving records from 101,773 cows in 3065 herds, with carcass information for 48,875 progeny, and found that sires ranked highly on the DBI produced heavier and better-conformed carcasses than sires chosen mainly for calving performance, with an estimated 3–5% increase in dairy-herd profitability under the assumptions of the analysis. The figure is specific to Irish production and market conditions and is not a guaranteed return elsewhere.

Semen allocation is therefore a whole-herd optimization problem in which expected calf revenue and semen cost are weighed against replacement balance, reproductive performance, and long-term genetic objectives. Because calf prices, semen costs, and fertility change over time, herd-specific decision-support models and sensitivity analyses are more useful than fixed recommendations.

### 5.3. Reproductive Performance and Herd Economics

Reproductive performance determines how many matings are available for beef semen, how many replacement heifers are produced, and how many calves reach the market, so semen allocation cannot rest on the expected value of crossbred calves alone. Cabrera [39] showed that the outcome of alternative allocation strategies depends strongly on reproductive performance and market conditions, and that ICOSC, being a partial indicator, omits production, health, labor, feed, and downstream rearing costs.

Reproductive risk also varies with sire breed. Basiel et al. [7] used 75,256 lactation records from 39,249 cows in 10 US herds to evaluate dystocia, stillbirth, GL, early-lactation health, early culling, and subsequent milk production: the probability of stillbirth was higher for calves sired by crossbred beef bulls than for Holstein-sired calves (5% vs. 2%), whereas dystocia risk, early-lactation clinical events, early culling, and milk production did not differ among sire-breed groups. Reproductive outcomes therefore have to be evaluated by sire breed and by specific trait rather than assumed uniform across beef-sired matings.

An economic assessment of BoD programs consequently has to integrate conception and pregnancy rates, pregnancy loss, stillbirth, dystocia, GL, replacement-heifer requirement, semen cost, and the market value of saleable calves, since these variables act at different stages and are not interchangeable. A higher calf price can be offset by lower reproductive efficiency, additional replacement cost, or losses at calving, and a longer GL has to be read against calving interval, cow productivity, and herd management rather than in isolation.

The main limitation of the evidence is that reproductive and economic outcomes are usually assessed separately or under model-specific assumptions. Joint herd-level analysis with sensitivity analysis across reproductive performance, sire-breed characteristics, replacement policy, and calf markets would separate partial indicators such as ICOSC from whole-farm profitability, and would need to be calibrated to the production system, since reproductive performance, replacement strategy, and calf marketing differ between confinement and pasture-based herds.

## 6. Carbon Footprint and Sustainability

### 6.1. Life Cycle Assessment and Environmental Efficiency of Integrated Dairy-Beef Systems

Life cycle assessment (LCA) quantifies emissions and resource use across defined production stages and system boundaries, and its application to BoD systems requires care because milk and beef are joint outputs of dairy production. Allocating burdens between them can change the result substantially: impacts may be expressed per kilogram of fat- and protein-corrected milk (FPCM), liveweight, carcass weight, or a combined milk-and-beef functional unit. Comparisons between systems are meaningful only when functional unit, system boundary, and allocation procedure are stated explicitly, since a difference in any of the three can reverse the ranking.

The environmental argument for BoD integration is that it produces additional beef within an existing dairy system and may reduce the need for dedicated suckler-cow production. That argument does not by itself establish a reduction in absolute greenhouse gas (GHG) emissions, nor is any such reduction attributable to beef-sire genetics alone. The outcome depends on the quantity of saleable beef produced, growth and survival, finishing duration, feed requirements, and the allocation of dairy-system burdens. Faster growth can lower emissions intensity by spreading maintenance emissions over more kilograms of beef even when emissions per animal are unchanged.

Evidence from New Zealand illustrates the scale that integration can reach. An AgResearch [43] LCA reported carbon footprints 32–48% below the national conventional-beef average for dairy-beef steers finished rapidly and processed at 10–18 months of age, with emissions expressed as kilograms of carbon dioxide equivalents (CO_2_-eq) per kilogram of finished liveweight and including animal production, input generation, transport, and other upstream processes. These are scenario-specific results from a technical report, obtained under rapid finishing, and they do not establish that comparable reductions would occur in slower-growing, pasture-only pathways.

Methodological choices matter as much as production data. Mazzetto et al. [44] showed, with an expanded-boundary LCA of Costa Rican cattle systems, that rankings changed when milk and beef were assessed jointly rather than milk alone: global-warming impact shifted once beef output and the suckler-beef production it displaces were included. The study compared dual-purpose and specialized systems rather than BoD strategies, so its contribution is methodological.

Throughout this review, environmental efficiency denotes GHG emissions per kilogram of saleable product, on a stated basis (liveweight, carcass weight, or FPCM), and not emissions per animal or per hectare. BoD assessments also need to separate attributional LCA, which assigns burdens to a defined product system, from consequential or expanded-boundary approaches that capture changes across interconnected dairy and beef systems. Where data permit, land occupation, eutrophication, acidification, and resource use are reported alongside the carbon footprint, because a reduction in GHG intensity can coexist with greater land demand.

### 6.2. From Grazing to Finishing: Management Pathways Shaping Environmental Efficiency in Dairy-Beef Systems

Environmental performance depends on genotype, nutrition, growth trajectory, and management across the cycle, so assessments restricted to a single phase or to final carcass performance miss the cumulative consequences of alternative grazing-to-finishing pathways [45].

Early-life nutrition and pasture quality carry that effect forward. Burggraaf et al. [46] evaluated dairy-beef heifer calves under different rearing diets and post-weaning pasture-quality treatments: calves offered higher-quality pasture between 3 and 7 months of age reached 31 kg more liveweight at 7 months than calves on lower-quality pasture, the advantage largely persisted to slaughter, and modeling indicated that improved pasture quality could reduce methane emissions and nitrate leaching by shortening the time to reach slaughter weight. The benefit comes from the forage resource rather than from the animal alone, and its size depends on the pathway and its feed inputs.

Slaughter age and functional unit interact in the same way. Herron et al. [45] compared four pasture-based suckler-beef systems by LCA and found the lowest impacts, across the categories and functional units assessed, in the system targeting slaughter at 20 months; the effects of supplementation and slaughter age were not uniform across comparisons, and breed rankings changed depending on whether impacts were expressed per kilogram of liveweight gain or of meat-weight gain. The study did not evaluate BoD cattle, but it shows that finishing strategy, carcass characteristics, and functional-unit definition have to be read together in pasture-based production.

Methane-mitigation technologies can complement gains in animal performance, but their effectiveness depends on diet and delivery. In beef cattle on a forage-based diet, Alemu et al. [47] reported that 3-nitrooxypropanol (3-NOP) reduced daily methane emissions by 22.4% and methane yield by 22.0% without affecting dry-matter intake or apparent total-tract nutrient digestibility. That is a controlled-feeding outcome and does not establish equivalent reductions under unrestricted grazing, where intake and additive delivery are harder to control. Red algae show larger effects under experimental conditions—Roque et al. [48] reported reductions exceeding 80% in beef steers supplemented with *Asparagopsis taxiformis*—with consistent intake, compound stability, safety, supply, and cost still unresolved for pasture systems. Such technologies complement improved performance and feeding management; they do not replace whole-system assessment.

Soil organic carbon requires the same discipline. Grasslands store carbon, but the magnitude and direction of change depend on climate, soil properties, vegetation, grazing intensity, and management history. McSherry and Ritchie [49] documented substantial variation in soil-carbon responses to grazing in a global meta-analysis; Abdalla et al. [50] found that grazing-intensity effects differed among climatic regions and grassland types; and Jordon et al. [51] emphasized the context-dependence of storage and the uncertainty surrounding its capacity to offset livestock emissions. Soil carbon cannot be counted against enteric methane or nitrous oxide without site-specific evidence, consistent accounting boundaries, and data on persistence.

These trade-offs are concrete in temperate pasture regions such as southern Chile, where perennial ryegrass (*Lolium perenne* L.) and other forage species support dairy and beef production. Forage availability and nutritive value vary seasonally, and performance depends on pasture composition, grazing management, stocking rate, supplementation, and finishing objectives. Pasture-based systems reduce dependence on purchased concentrates and some upstream emissions, whereas slower growth raises cumulative maintenance and enteric emissions per kilogram of beef; intensive finishing shortens the cycle while increasing reliance on external feed. Neither is inherently superior without whole-system comparison.

For BoD systems the question is whether the efficiencies associated with dairy-origin calves persist under specific grazing and finishing conditions. Pasture-only, pasture-plus-supplementation, and confinement-finishing pathways need comparison on growth, mortality, slaughter age, carcass output, and upstream inputs; evidence from other temperate regions is a starting point, not a substitute for regional data.

### 6.3. Beyond Emissions Intensity: Dairy-Beef Integration in the Latin American Sustainability Context

The environmental implications of BoD in Latin America have to be read against regional cattle production, land use, and production efficiency. Livestock systems differ widely in climate, forage resources, herd structure, productivity, and management intensity, so regional outcomes cannot be inferred from a single production model. Velarde-Guillén et al. [52], reviewing carbon-footprint assessments of Latin American dairy systems, reported substantial variation among systems and showed how strongly the production context shapes estimates of emissions per kilogram of FPCM. That evidence concerns milk rather than BoD, but it establishes the need for regionally appropriate data and consistent LCA methods.

In temperate dairy regions of South America, including southern Chile, BoD can increase beef output from calves generated within existing dairy herds and use established farm infrastructure and forage resources. The size of that benefit depends on the proportion of beef-sired calves successfully reared, their growth and survival, finishing duration, and the quantity and quality of beef produced; it also depends on the burdens assigned to milk and beef and on whether additional beef production displaces or merely supplements output from specialized beef systems. None of these questions is settled by comparing emissions intensity alone.

The regional context frames both sides of that balance: Argentina counted 7069 dairy farms in its 2018 agricultural census [53], and in the inventories compiled by FAOSTAT enteric fermentation from cattle remains the largest single source of agricultural emissions across the main South American producing countries [54]. Beef output that grows through better use of calves already born in dairy herds therefore acts on emissions per kilogram of product rather than on the size of the national herd.

Capturing the opportunity requires local evaluation rather than transferred parameters. Selection indices calibrated for temperate South American systems would need regional genetic evaluations, feed resources, calf and carcass prices, labor costs, and production constraints, and would combine breeding values and economic weights for calving ease (CE), GL, growth, carcass merit, feed efficiency, and survival. Genotype-by-environment interaction also needs testing, because sire rankings derived from intensive finishing systems are not automatically valid under pasture-based management. Until that evidence exists, balanced sire selection with system-specific economic assessment is more defensible than assuming that one beef breed is optimal everywhere.

The central limitation is the scarcity of LCA studies of BoD cattle finished on permanent pasture under temperate Latin American conditions. Farm-level production data combined with consistent LCA methods would allow alternative sire breeds and grazing-to-finishing pathways to be compared, reporting absolute GHG emissions and emissions intensity, stating whether the functional unit is liveweight or carcass weight, describing the treatment of milk and beef co-products, and quantifying trade-offs in land use, feed inputs, and soil-carbon change, with sensitivity analyses on slaughter age, growth rate, pasture productivity, supplementation, and allocation method. Until such comparisons exist, BoD is a promising route to closer dairy-beef integration rather than a demonstrated carbon-mitigation strategy, and its contribution to sustainability will rest on measured whole-system resource efficiency supported by regional evidence.

## 7. Animal Welfare in Beef-on-Dairy Systems

Welfare in BoD systems has to be assessed across the whole chain, from calving and neonatal care through rearing, transport, marketing, finishing, and slaughter, because a risk introduced at one stage is not necessarily corrected later. At birth, sire selection influences gestation length, birth weight, and calving difficulty, with consequences for neonatal survival and for the dam. The magnitude of that risk depends on the individual sire, the dam, and the management environment rather than on the beef breed label. The evidence base is also uneven: neonatal care and transport are comparatively well documented, whereas rearing, finishing, and slaughter of BoD cattle have been assessed mainly through carcass and meat-quality outcomes rather than through welfare outcomes.

During the neonatal period, timely colostrum administration and adequate transfer of passive immunity are the main determinants of calf health. In a cross-sectional study of 15 commercial dairy farms in Indiana and Michigan, Kern et al. [55] found no difference in passive immunity between purebred dairy and dairy × beef calves managed within the same systems, whereas calves receiving their first colostrum within 1 h of birth had better passive-immunity outcomes than calves first fed between 1 and 6 h after birth. Management timing carried more weight than crossbred status.

The value assigned to different calf categories may nonetheless shape the resources devoted to their care. Felix et al. [56], as reported by Kern et al. [55], found that fewer than 60% of surveyed dairy producers in the northeastern United States routinely administered colostrum to beef × dairy calves. That is not evidence that terminal crossbred calves are systematically neglected, but it shows that neonatal protocols are not applied uniformly across calf categories, which is the condition under which differential care would arise.

Neonatal welfare therefore rests on calving supervision, prompt colostrum, navel care, adequate nutrition, and early disease detection. Because the higher market value of crossbred calves can create an incentive to invest in their care without guaranteeing it, welfare assessment needs measurable outcomes—stillbirth, neonatal mortality, morbidity, serum indicators of passive immunity, and dystocia incidence—rather than calf value as a proxy for care quality.

### 7.1. Transport, Marketing and Supply-Chain Challenges

The risks extend beyond the farm of birth. Early separation, transport, commingling, handling, feed restriction, and changes in housing and feeding impose multiple stressors on young calves, and their capacity to cope depends on health, nutritional status, age, and preparation before departure; fitness for transport is therefore a question of clinical condition and physiological readiness, not of journey duration alone.

In 640 male dairy calves in western Canada, Wilson et al. [57] identified at least one clinical abnormality before transport in 37% of animals, with navel disease, low body weight, and depressed behavior associated with poorer subsequent health; during the first two weeks after transport, 23% were treated for diarrhea, 44% for bovine respiratory disease, and 4% died. England et al. [58] assessed 420 bob veal calves arriving at an Ohio abattoir and found 23.4% with failure of passive transfer, 68.6% dehydrated, and 73.9% hypoglycemic. Those calves were destined for bob veal, so the figures are not prevalence estimates for BoD calves entering conventional beef systems, but they show how inadequate neonatal care and subsequent marketing combine.

Schuenemann and Piñeiro [59] evaluated 392,064 preweaned dairy and dairy-beef crossbred calves transported for 0.5–24 h. Mortality by weaning was 2.49% overall and differed among transport-duration groups, and failure of passive transfer, calf disease, dystocia, gestation length, sex, and season were also associated with survival. The study was observational and calf categories were not distributed equally across durations, so the differences are not evidence that a given journey length caused mortality; they indicate that transport outcomes have to be read alongside pre-transport health and management. Experimental evidence points the same way: in 82 male Holstein calves, Pisoni et al. [60] found that limited colostrum at birth and feed restriction during simulated marketing were associated with poorer recovery after transport, including reduced body-weight and feed-intake recovery and less favorable health indicators.

Appeasing-substance studies in weaned feedlot cattle [61,62] report improvements in selected physiological, health, or performance outcomes, with responses varying by design and outcome; they are supplementary measures, not substitutes for adequate nutrition, low-stress handling, transport conditions, and health management, and evidence from weaned feedlot cattle does not transfer directly to neonatal BoD calves.

The quantitative evidence is summarized in Table 2.

### 7.2. Sex, Genetic Background, and Production-System Considerations

Welfare outcomes reflect interactions among calf sex, sire genetics, dam characteristics, and management. Male dairy calves have historically received less intensive neonatal care than replacement heifers in some systems, but that is a consequence of management priorities rather than a biological difference in welfare requirements, and colostrum management can support adequate passive immunity in both dairy and dairy × beef calves.

Sire selection acts on perinatal welfare through gestation length, birth weight, and calving difficulty. Using Irish National Cattle Database records for up to 977,037 progeny, Berry et al. [1] reported odds of dystocia relative to Holstein-Friesian sires of 1.43 for Angus and 4.77 for Belgian Blue before adjustment for dam and calf merit, which are breed-level contrasts rather than fixed effects independent of individual sire genetics. Garcia et al. [63] analyzed more than 1.2 million calving records from beef-on-dairy matings and documented genetic variation in calving ease, supporting the use of calving-ease estimated breeding values alongside carcass and growth traits. Basiel et al. [7] found a higher probability of stillbirth among calves sired by crossbred beef bulls than among Holstein-sired calves (5% vs. 2%) with no significant difference in dystocia, and longer gestations whose magnitude depended on sire breed.

Selection that balances carcass value with calving ease, gestation length, and welfare-relevant traits is therefore more defensible than selection on breed averages, and direct and maternal breeding values for calving difficulty are most informative when sires are mated to heifers or to cows with a history of difficult calving. In Irish pasture-based Holstein-Friesian herds, calving assistance and dystocia are associated with parity, calf sex, sire characteristics, and previous calving history [64], although that evidence concerns dairy calving broadly rather than BoD calves under grazing. Calving timing and location, weather exposure, labor availability, opportunities for early observation, and access to colostrum can all modify the risks that genotype imposes.

## 8. Integral Evaluation of Beef-on-Dairy Systems and Research Priorities for Pasture-Based Production

Evidence published since 2016 supports a narrow claim: BoD crossbreeding increases the value of dairy-origin calves and alters selected carcass characteristics. Using Irish national cattle records, Berry et al. [1] documented differences among beef sire breeds in calving performance, calf sale price, and carcass traits. The size of those differences depends on genetic merit, management, production system, and market conditions, and improvements in individual traits do not translate automatically into greater whole-farm profit or lower emissions. BoD is therefore better treated as an integrated production strategy than as a way of raising calf-sale value.

The central limitation of the literature is that integration is missing: large commercial datasets have quantified breed differences in calf value, calving outcomes, and carcass characteristics, but few studies have linked those outcomes to calf survival and health, feed use, finishing cost, whole-farm profit, and environmental impact within the same production system.

The gap matters because pasture-based systems differ from confinement in seasonal forage availability and quality, nutrient intake, growth trajectory, supplementation, finishing duration, and slaughter endpoint. These differences can change the expression of economically relevant traits and the suitability of individual sires, so genotype × environment interaction remains an open question for growth, feed efficiency, carcass composition, and profitability; sire rankings obtained in confined systems are a hypothesis to be tested, not a result to be transferred.

An integrated evaluation also separates biological outcomes from their economic and environmental consequences. Higher calf prices or heavier carcasses can be offset by longer finishing, additional supplementation, or higher production cost, and greater output per animal does not imply lower emissions per kilogram. Calving difficulty, stillbirth, neonatal morbidity, passive immunity, and transport-related health belong in the same assessment, because they affect both welfare and farm performance.

Six questions follow, each stated so that it can be answered with a measurable outcome rather than with a direction of effect: (1) Does the ranking of sires for calving ease and perinatal survival change between confinement and temperate pasture, when the same sires are progeny-tested in both environments? (2) What is the difference in RFI between BoD and pure Holstein cattle at pasture, expressed per unit of dry-matter intake rather than per kilogram of liveweight? (3) Does the slaughter age that minimizes CO_2_-eq per kilogram of carcass coincide with the age that maximizes net return per hectare? (4) Does pre-weaning mortality of BoD calves differ from that of Holstein calves born in the same herd, once colostrum management and calving supervision are accounted for? (5) How much of the value of the Irish DBI is recovered in South American pasture-based systems? (6) What proportion of the economic benefit of BoD depends on the calf price premium rather than on biological efficiency?

Answering them requires longitudinal, farm-level designs that connect individual sire and dam characteristics to reproductive performance, calf survival and health, growth, pasture utilization, finishing duration, slaughter weight, carcass value, and production cost, combined with LCA on stated functional units, boundaries, and allocation procedures, and with sensitivity scenarios for pasture availability, supplementation cost, calf prices, and replacement requirement. Only that structure separates genetic effects from those of management and of the wider production system.

The agenda is most pressing in temperate dairy regions of South America, including southern Chile, where pasture growth patterns, seasonal feed deficits, finishing pathways, labor constraints, calf-marketing infrastructure, and prices differ from those of Ireland or New Zealand. Evidence from those regions is a reference point, not a local result.

The value of BoD does not come from replacing dairy semen with beef semen. It comes from the combination of genetic merit, reproductive performance, nutrition, market incentives, welfare, and environmental outcomes within a given system, and the operative question is under which conditions that combination is favorable.

## 9. Conclusions

The evidence reviewed supports a narrow set of conclusions and leaves a wider set open. Benefits of BoD crossbreeding are most consistently documented for calf value and selected carcass traits, whereas feed efficiency, whole-farm profitability, environmental performance, and animal welfare depend on genetic merit, reproductive performance, management, and production-system conditions.

Calf value and carcass merit respond to sire selection, but not uniformly. Breed differences in calf price and carcass weight are large enough to matter commercially—a €125.4 spread between the highest- and lowest-valued beef breeds in Irish records—yet they vary with market specification, calf sex, and production system, and breed means are not sire values.

Whole-farm profitability is not implied by trait improvement. Higher calf prices and heavier carcasses can be offset by longer finishing, additional supplementation, lower reproductive efficiency, or higher replacement cost, so partial indicators such as ICOSC or carcass value per animal do not substitute for a whole-farm budget.

Reproductive and perinatal outcomes are trait-specific rather than breed-specific. Stillbirth, dystocia, and gestation length differ among sire breeds in different directions and magnitudes, and individual sire merit explains part of the variation that breed labels conceal, so calving-ease breeding values carry information that breed identity does not.

Environmental performance depends on the accounting as much as on the animal. Reductions in emissions intensity of 32–48% have been reported for rapidly finished dairy-beef steers in New Zealand, but those figures rest on a defined functional unit, system boundary, and finishing pathway, and they do not transfer to slower pasture-based systems without local measurement.

The main limitation remains the scarcity of integrated evaluations under permanent pasture. Findings from Ireland, New Zealand, and other regions are reference points rather than transferable results, and their applicability to pasture-based dairy systems depends on forage availability, seasonal feed deficits, growth trajectories, finishing pathways, and market conditions—conditions that differ in temperate South America, including southern Chile.

BoD is therefore a context-dependent strategy rather than a universal replacement for conventional dairy breeding. Its value lies in the coordinated use of genetics, reproductive management, and production-system objectives, and establishing the conditions under which that coordination produces reliable net returns while maintaining welfare and environmental performance remains the central task.

## Figures and Tables

**Figure 1:**
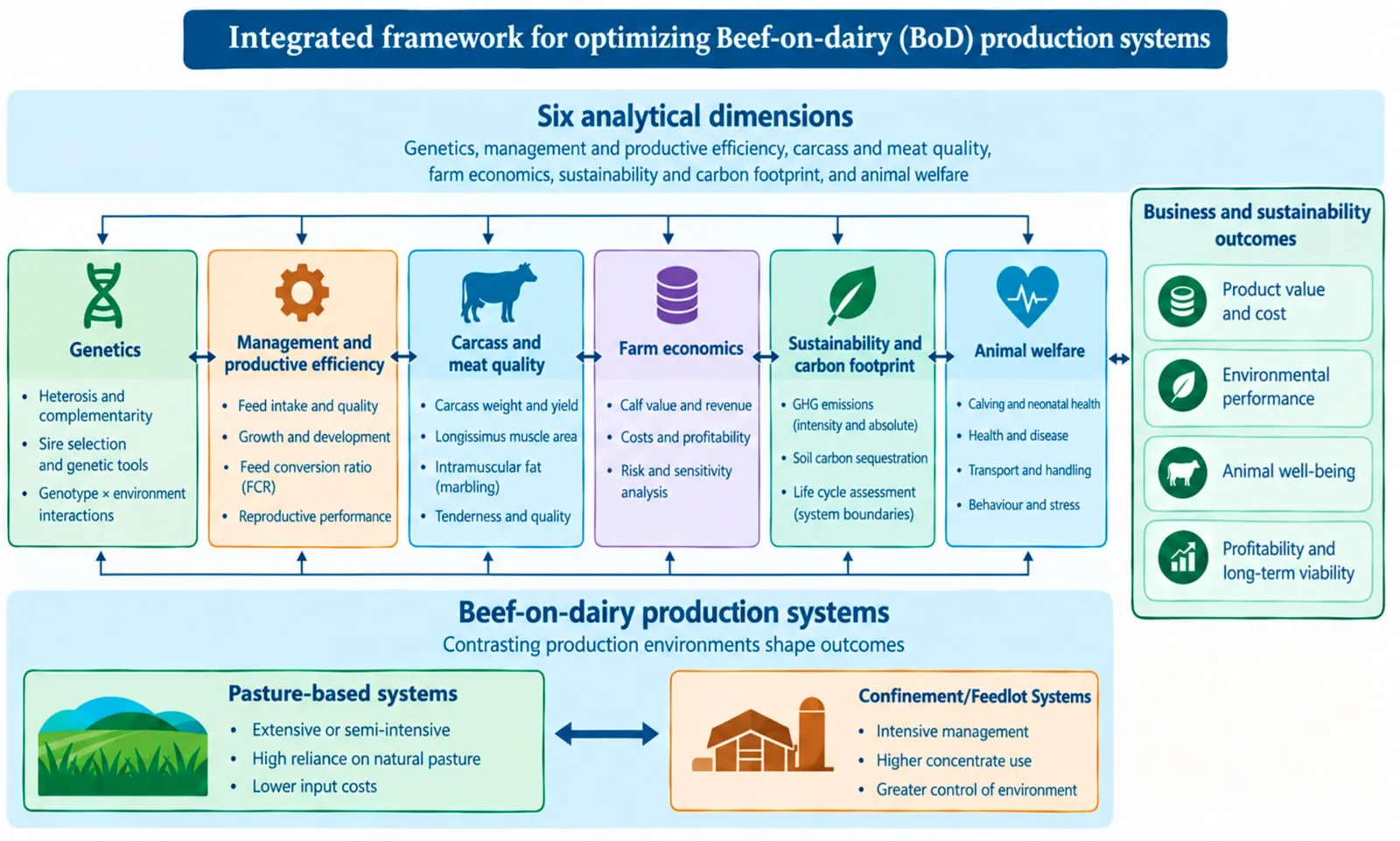
Conceptual framework for beef-on-dairy (BoD) production systems. Six analytical dimensions—genetics (sire selection, crossbreeding, genotype-by-environment interaction); management and productive efficiency (feed intake, growth, feed conversion); carcass and meat quality (carcass yield, longissimus muscle area, intramuscular fat, tenderness); farm economics (calf value, revenue, production costs, profitability); sustainability (greenhouse gas emissions, soil carbon sequestration, life cycle assessment); and animal welfare (neonatal health, disease, transport, handling, behavior, stress)—are evaluated within two contrasting production contexts, pasture-based and confinement/feedlot. The framework is conceptual: it identifies interactions among dimensions and contexts and does not imply a fixed sequence or uniform effects across production systems.

**Table 1:** Quantitative evidence on growth performance and carcass traits of beef-on-dairy (BoD) progeny according to sire breed.

Study and System	Sire Breed	*n*	ADG (kg/day)	HCW (kg)	DP (%)	Marbling Score	WBSF (kg)
[7]	Angus	47	1.76 ^a^	392	61.7	4.82 ^ab^	3.82 ^c^
	Red Angus	28	1.62 ^bc^	382	61.9	5.03 ^a^	4.14 ^abc^
	Charolais	92	1.73 ^ab^	381	60.7	4.71 ^ab^	4.30 ^ab^
	Hereford	19	1.66 ^abc^	371	60.2	4.61 ^abc^	3.83 ^bc^
	Wagyu	11	1.39 ^d^	355	61.1	4.59 ^abc^	3.93 ^abc^
	Limousin	18	1.55 ^cd^	369	61.0	4.14 ^c^	3.70 ^bc^
	Simmental/SimAngus	47	1.68 ^ab^	392	61.4	4.50 ^bc^	4.51 ^a^
[1]	Angus	Breed-specific *n* not provided	Not reported	327.1	Not reported		
	Belgian Blue			363.2			
	Holstein-Friesian			322.4			

ADG, average daily gain; DP, dressing percentage; HCW, hot carcass weight; WBSF, Warner–Bratzler shear force. Basiel et al. (2024) evaluated 262 beef × Holstein steers from 10 Pennsylvania dairy farms; *n* indicates the number of steers per sire-breed group, and Simmental and SimAngus were pooled. Different superscript letters within a column indicate differences among sire-breed groups (*p* < 0.05). HCW and DP did not differ among groups (NS), nor did the gain-to-feed ratio (NS); marbling score and WBSF differed among groups (*p* < 0.05). Marbling score is expressed on the scale used by Basiel et al. (2024). Berry et al. (2023) reported unadjusted mean carcass weights from Irish national records, which did not control for age at slaughter or carcass fat. The US data represent intensive feedlot finishing and cannot be extrapolated directly to permanent-pasture systems.

**Table 2:** Calving performance, neonatal health, and survival in beef-on-dairy (BoD) production.

Study	Population/Sample	Indicator	Quantitative Finding
[7]	75,256 lactations; 10 US herds	Stillbirth	5% for beef-sired calves vs. 2% for Holstein-sired calves.
[63]	231,000 first-lactation records; 1.2 million across three lactations	Difficult calving	Approximately 15% incidence; direct and maternal genetic variation was identified.
[55]	Calves from 15 dairy farms	Poor passive immunity	Mean prevalence of 27.3%; no clear difference between dairy and dairy × beef calves.
[59]	392,064 preweaned calves	Mortality before weaning	Overall mortality of 2.49% (9760 deaths)
[57]	640 male dairy calves	Clinical abnormalities before transport	37% had at least one clinical abnormality. Contextual evidence, not a BoD-specific cohort.

Studies differ in population and design. Results from general dairy-calf populations should not be interpreted as BoD-specific effects.

## Data Availability

No new data were created or analyzed in this study. Data sharing is not applicable to this article.

## Abbreviations

The following abbreviations are used in this manuscript:

ADG	Average daily gain
BoD	Beef on Dairy
CE	Calving ease
DBI	Dairy Beef Index
DMI	Dry matter intake
DOF	Days on feed
DP	Dressing percentage
FCR	Feed conversion ratio
FPCM	Fat- and Protein-Corrected Milk
G	Gain-to-feed ratio
GHG	Greenhouse gases
GL	Gestation length
ICOSC	Income Over Semen Cost
IMF	Intramuscular fat
IPCC	Intergovernmental Panel on Climate Change
LCA	Life Cycle Assessment
PUFA	Polyunsaturated fatty acids
RFI	Residual feed intake
WBSF	Warner–Bratzler Shear Force

## References

[1] Berry D.P., Twomey A., Ring S. (2023). Mean Breed Performance of the Progeny from Beef-on-Dairy Matings. J. Dairy Sci..

[2] Berry D.P. (2021). Invited Review: Beef-on-Dairy—The Generation of Crossbred Beef × Dairy Cattle. J. Dairy Sci..

[3] Basiel B.L., Felix T.L. (2022). Board Invited Review: Crossbreeding Beef × Dairy Cattle for the Modern Beef Production System. Transl. Anim. Sci..

[4] Silva Neto J.B., Mota L.F.M., Londoño-Gil M., Schmidt P.I., Rodrigues G.R.D., Ligori V.A., Arikawa L.M., Magnabosco C.U., Brito L.F., Baldi F. (2024). Genotype-by-environment Interactions in Beef and Dairy Cattle Populations: A Review of Methodologies and Perspectives on Research and Applications. Anim. Genet..

[5] Berry D.P., Amer P.R., Evans R.D., Byrne T., Cromie A.R., Hely F. (2019). A Breeding Index to Rank Beef Bulls for Use on Dairy Females to Maximize Profit. J. Dairy Sci..

[6] Ahmed R.H., Schmidtmann C., Mugambe J., Thaller G. (2024). Factors Influencing Calving Difficulty and Gestation Length in Dairy Cows Inseminated with Beef Sires. Animal.

[7] Basiel B.L., Barragan A.A., Felix T.L., Dechow C.D. (2024). The Impact of Beef Sire Breed on Dystocia, Stillbirth, Gestation Length, Health, and Lactation Performance of Cows That Carry Beef × Dairy Calves. J. Dairy Sci..

[8] Jaborek J.R., Carvalho P.H.V., Felix T.L. (2023). Post-Weaning Management of Modern Dairy Cattle Genetics for Beef Production: A Review. J. Anim. Sci..

[9] Mugambe J., Schmidtmann C., Hidalgo J., Ahmed R., Thaller G. (2026). Genetic Evaluation of Beef Sires Using a Beef-on-Dairy Crossbred Reference Population. J. Anim. Breed. Genet..

[10] Ahmed R.H., Schmidtmann C., Mugambe J., Thaller G. (2025). Genomic-Based Genetic Parameters and Genome-Wide Association Studies for Productive and Reproductive Traits in Beef-on-Dairy Crossbreds. Front. Genet..

[11] Ashfield A., Wallace M., Crosson P. (2014). Economic Comparison of Pasture Based Dairy Calf-to-Beef Production Systems under Temperate Grassland Conditions. Int. J. Agric. Manag..

[12] Finneran E., Crosson P., O’Kiely P., Shalloo L., Forristal D., Wallace M. (2010). Simulation modelling of the cost of producing and utilising feeds for ruminants on Irish farms. J. Farm Manag..

[13] Gregory K.E., Cundiff L.V. (1980). Crossbreeding in Beef Cattle: Evaluation of Systems. J. Anim. Sci..

[14] Dillard E.U., Rodriguez O., Robison O.W. (1980). Estimation of Additive and Nonadditive Direct and Maternal Genetic Effects from Crossbreeding Beef Cattle. J. Anim. Sci..

[15] Knapp B.W., Pahnish O.F., Urick J.J., Brinks J.S., Richardson G.V. (1980). Preweaning and Weaning Heterosis for Maternal Effects of Beef × Beef and Beef × Dairy Crosses. J. Anim. Sci..

[16] VanRaden P.M., Tooker M.E., Chud T.C.S., Norman H.D., Megonigal J.H., Haagen I.W., Wiggans G.R. (2020). Genomic Predictions for Crossbred Dairy Cattle. J. Dairy Sci..

[17] Wientjes Y.C.J., Bijma P., Calus M.P.L. (2020). Optimizing Genomic Reference Populations to Improve Crossbred Performance. Genet. Sel. Evol..

[18] Lindtke D., Lerch S., Morel I., Neuditschko M. (2024). Assessment of Genome Complementarity in Three Beef-on-Dairy Crossbreds Reveals Sire-Specific Effects on Production Traits with Comparable Rates of Genomic Inbreeding Reduction. BMC Genom..

[19] Maciel I.C.F., Schweihofer J.P., Fenton J.I., Hodbod J., McKendree M.G.S., Cassida K., Rowntree J.E. (2021). Influence of Beef Genotypes on Animal Performance, Carcass Traits, Meat Quality, and Sensory Characteristics in Grazing or Feedlot-Finished Steers. Transl. Anim. Sci..

[20] Berry D.P., Ring S.C. (2020). Observed Progeny Performance Validates the Benefit of Mating Genetically Elite Beef Sires to Dairy Females. J. Dairy Sci..

[21] Carter R.E., Emenheiser J.C., Zinn S.A., Govoni K.E., Felix T.L., Reed S.A. (2025). Effects of Milk Replacer Composition on Growth and Development of Beef × Dairy Crossbred Calves. Transl. Anim. Sci..

[22] Stypinski J.D., Plumski A.L., Ziegler D.M., Hansen W.P., Scott M.F., Salfer I.J. (2025). Impacts of Feeding Milk Replacer Containing 20% or 24% Protein and Fat on Growth and Feed Efficiency of Beef × Dairy Crossbred Calves. JDS Commun..

[23] Grote A., Genther-Schroeder O., Wilson B.K., Wistuba T., Beck P. (2025). Plane of Nutrition through 92 Days of Age in Beef × Dairy Hybrid Steers Influences Subsequent Finishing Performance, Feed Efficiency, and Carcass Characteristics. Appl. Anim. Sci..

[24] Grote A., Fanning T., DeVuyst E.A., Grigsby Z., Crosswhite J., Beck P. (2025). A Commercial Scale Evaluation of the Effects of Post-Weaning Management System of Dairy × Beef Hybrid Steers Compared to Native Beef Steers on Performance, Carcass Characteristics, and Net Returns. Transl. Anim. Sci..

[25] Husz T.C., Word A.B., Karr K.J., Holland B.P., Lawrence T.E., Perkins T.L., Hutcheson J.P., Walter L.J. (2024). Effects of Days on Feed on Performance and Carcass Characteristics of Crossbred Beef × Dairy Heifers. Appl. Anim. Sci..

[26] Schmaltz C.S., Schroeder T.C., Walter L.J., Hutcheson J.P. (2024). Economic Returns and Market Risk of Extending Days on Feed for Beef × Dairy Cross Heifers. Appl. Anim. Sci..

[27] Crawford G.I., Walter L.-A.J., Word A.B., Karr K., Holland B.P., Hutcheson J.P., Nichols W.T., Streeter M.N., Nuttelman B.L. (2024). 59 Effects of Serial Harvest on Growth and Carcass Characteristics of Beef x Dairy Crossbred Steers. J. Anim. Sci..

[28] Morales Gómez J.F., Antonelo D.S., Beline M., Pavan B., Bambil D.B., Fantinato-Neto P., Saran-Netto A., Leme P.R., Goulart R.S., Gerrard D.E. (2022). Feeding Strategies Impact Animal Growth and Beef Color and Tenderness. Meat Sci..

[29] Van Koevering M.T., Gill D.R., Owens F.N., Dolezal H.G., Strasia C.A. (1995). Effect of Time on Feed on Performance of Feedlot Steers, Carcass Characteristics, and Tenderness and Composition of Longissimus Muscles. J. Anim. Sci..

[30] Herd R.M., Arthur P.F. (2009). Physiological Basis for Residual Feed Intake. J. Anim. Sci..

[31] Pimentel-Concepción M., Jaborek J.R., Schweihofer J.P., Garmyn A.J., McKendree M.-G.-S., Bradford B.J., Hentschl A., Buskirk D.D. (2024). Growth Performance, Carcass Traits, and Feeder Calf Value of Beef × Holstein and Holstein Feedlot Steers. Appl. Anim. Sci..

[32] Rezagholivand A., Nikkhah A., Khabbazan M.H., Mokhtarzadeh S., Dehghan M., Mokhtabad Y., Sadighi F., Safari F., Rajaee A. (2021). Feedlot Performance, Carcass Characteristics and Economic Profits in Four Holstein-Beef Crosses Compared with Pure-Bred Holstein Cattle. Livest. Sci..

[33] Martín N., Schreurs N., Morris S., López-Villalobos N., McDade J., Hickson R. (2021). Sire Effects on Carcass of Beef-Cross-Dairy Cattle: A Case Study in New Zealand. Animals.

[34] Bittante G., Bergamaschi M., Qianlin N., Patel N., Toledo-Alvarado H., Cecchinato A. (2023). Veal and Beef Meat Quality of Crossbred Calves from Dairy Herds Using Sexed Semen and Semen from Double-Muscled Sires. Ital. J. Anim. Sci..

[35] Drachmann F.F., Johansen K., Kargo M., Buitenhuis A.J., Therkildsen M. (2025). Beef-on-Dairy: Current and Potential Meat Quality of Dairy-Based Beef Production with Culled Holstein Cows and Danish Blue × Holstein Crossbred Calves. Acta Agric. Scand. Sect. A—Anim. Sci..

[36] Ivanković A., Pećina M., Bittante G., Kelava Ugarković N., Konjačić M. (2025). Growth Performance, Carcass Characteristics, and Meat Quality in Beef × Holstein-Friesian Crossbred Bulls. Ital. J. Anim. Sci..

[37] Meier L.E., Golden B.L., Newman S., Spangler M.L. (2026). Development of Economic Selection Indexes for Beef-on-Dairy Systems and Implication for Sire Re-Ranking in U.S. Production Systems. Transl. Anim. Sci..

[38] CoBank (2025). Beef-on-Dairy Data Suggests Opportunity for Feedlots and Processors. CoBank Knowl. Exch..

[39] Cabrera V.E. (2022). Economics of Using Beef Semen on Dairy Herds. JDS Commun..

[40] Clasen J.B., Kargo M., Østergaard S., Fikse W.F., Rydhmer L., Strandberg E. (2021). Genetic Consequences of Terminal Crossbreeding, Genomic Test, Sexed Semen, and Beef Semen in Dairy Herds. J. Dairy Sci..

[41] Pereira J.M.V., Bruno D., Marcondes M.I., Ferreira F.C. (2022). Use of Beef Semen on Dairy Farms: A Cross-Sectional Study on Attitudes of Farmer Toward Breeding Strategies. Front. Anim. Sci..

[42] Ferrari V., Marusi M., Penasa M., Van Kaam J.B.C.H.M., Finocchiaro R., Cassandro M. (2024). A Tool to Optimise Dairy Herd Replacements Combining Conventional, Sexed, and Beef Semen. Ital. J. Anim. Sci..

[43] AgResearch (2024). Carbon Footprint of 10–18 Month-Old Dairy Beef Production Systems.

[44] Mazzetto A.M., Bishop G., Styles D., Arndt C., Brook R., Chadwick D. (2020). Comparing the Environmental Efficiency of Milk and Beef Production through Life Cycle Assessment of Interconnected Cattle Systems. J. Clean. Prod..

[45] Herron J., Curran T.P., Moloney A.P., McGee M., O’Riordan E.G., O’Brien D. (2021). Life Cycle Assessment of Pasture-Based Suckler Steer Weanling-to-Beef Production Systems: Effect of Breed and Slaughter Age. Animal.

[46] Burggraaf V.T., Craigie C.R., Muir P.D., Khan M.A., Thomson B.C., Knol F.W., Lowe K.A., Taukiri K.R., Staincliffe M., McDermott A. (2020). Effect of Rearing Diet and Early Post-Weaning Pasture Quality on the Life-Time Growth, Meat Quality, Carcass Traits and Environmental Impact of Dairy-Beef Cattle. Livest. Sci..

[47] Alemu A.W., Gruninger R.J., Zhang X.M., O’Hara E., Kindermann M., Beauchemin K.A. (2023). 3-Nitrooxypropanol Supplementation of a Forage Diet Decreased Enteric Methane Emissions from Beef Cattle without Affecting Feed Intake and Apparent Total-Tract Digestibility. J. Anim. Sci..

[48] Roque B.M., Venegas M., Kinley R.D., De Nys R., Duarte T.L., Yang X., Kebreab E. (2021). Red Seaweed (Asparagopsis Taxiformis) Supplementation Reduces Enteric Methane by over 80 Percent in Beef Steers. PLoS ONE.

[49] McSherry M.E., Ritchie M.E. (2013). Effects of Grazing on Grassland Soil Carbon: A Global Review. Glob. Change Biol..

[50] Abdalla M., Hastings A., Chadwick D.R., Jones D.L., Evans C.D., Jones M.B., Rees R.M., Smith P. (2018). Critical Review of the Impacts of Grazing Intensity on Soil Organic Carbon Storage and Other Soil Quality Indicators in Extensively Managed Grasslands. Agric. Ecosyst. Environ..

[51] Jordon M.W., Buffet J.-C., Dungait J.A.J., Galdos M.V., Garnett T., Lee M.R.F., Lynch J., Röös E., Searchinger T.D., Smith P. (2024). A Restatement of the Natural Science Evidence Base Concerning Grassland Management, Grazing Livestock and Soil Carbon Storage. Proc. R. Soc. B Biol. Sci..

[52] Velarde-Guillén J., Arndt C., Gómez C.A. (2022). Carbon Footprint in Latin American Dairy Systems. Trop. Anim. Health Prod..

[53] Instituto Nacional de Estadística y Censos (2019). Censo Nacional Agropecuario 2018: Resultados Definitivos.

[54] FAO (2024). FAOSTAT: Emissions from Agrifood Systems.

[55] Kern J., Jorgensen M.W., Boerman J.P., Erasmus M., Johnson J.S., Pempek J.A. (2025). Differences in Colostrum Management and Transfer of Passive Immunity between Purebred Dairy and Dairy × Beef Crossbred Calves in Indiana and Michigan. Transl. Anim. Sci..

[56] Felix T.L., Emenheiser J.C., Govoni K.E., Zinn S.A., Reed S.A. (2023). Survey of the Use of Beef Semen in Dairy Herds in Pennsylvania and Nearby States. Transl. Anim. Sci..

[57] Wilson D.J., Stojkov J., Renaud D.L., Fraser D. (2020). Risk Factors for Poor Health Outcomes for Male Dairy Calves Undergoing Transportation in Western Canada. Can. Vet. J. Rev. Vet. Can..

[58] England Z.A., Maggard H.L., Fisher A.D., Roadknight N.W., Pempek J.A. (2023). Condition of Bob Veal Calves on Arrival at an Abattoir in Ohio. Anim. Welf..

[59] Schuenemann G.M., Piñeiro J.M. (2025). Short- and Long-Distance Transport: Health, Survival and Growth of Preweaned Dairy and Dairy-Beef Cross Calves. J. Anim. Sci..

[60] Pisoni L., Devant M., Bassols A.M., Saco Y., Pato R., Pujols J., Marti S. (2023). The Effects of Colostrum Consumption and Feed Restriction during Marketing and Transportation of Male Dairy Beef Calves: Impact on Pre-Transport Nutritional Status and on Farm Recovery. J. Dairy Sci..

[61] Pickett A.T., Cooke R.F., De Souza I.S., Mackey S.J. (2024). Administering the Maternal Bovine Appeasing Substance Improves Overall Productivity and Health in High-Risk Cattle during a 60-d Feedlot Receiving Period. J. Anim. Sci..

[62] Kvamme K., Marques R.S., Alves Cruz V., Limede Cintra A., Ogg M.A., McCoski S.R., Posbergh C.J., Bradbery A.N., Mercadante V.R.G., Mackey S.J. (2024). Multiple Administrations of Bovine-Appeasing Substance during a 42-d Preconditioning Program Followed by Feedlot Receiving and Its Effects on Physiologic, Health, and Performance Responses of Feeder Cattle. J. Anim. Sci..

[63] Garcia A.O., Mikush A.A., Cole J.B., Tsuruta S., Misztal I., Guimarães S.E.F., Lourenco D. (2025). Genetic Background of Calving Ease in Beef-on-Dairy. J. Dairy Sci..

[64] Mee J.F., Berry D.P., Cromie A.R. (2011). Risk Factors for Calving Assistance and Dystocia in Pasture-Based Holstein–Friesian Heifers and Cows in Ireland. Vet. J..

